# Essential genes in thyroid cancers: focus on fascin

**DOI:** 10.1186/2251-6581-12-32

**Published:** 2013-07-01

**Authors:** Hilda Samimi, Majid Zaki dizaji, Mohsen Ghadami, Abolhasan Shahzadeh fazeli, Patricia Khashayar, Masoud Soleimani, Bagher Larijani, Vahid Haghpanah

**Affiliations:** 1 Science and Culture University, Tehran, Iran; 2 Endocrinology and Metabolism Research Center, Endocrinology and Metabolism Research Institute, Tehran University of Medical Sciences, 5th floor, Dr. Shariati Hospital, North Kargar Ave, Tehran, Iran; 3 Department of Medical Genetics, Tehran University of Medical Sciences, Tehran, Iran; 4 Department of Hematology, Faculty of Medical Science, Tarbiat Modares University, Tehran, Iran

**Keywords:** Thyroid cancer, Mutation, Rearrangement, P53, RAS, RET, BRAF, PPARγ, Fascin

## Abstract

Although thyroid cancers are not among common malignancies, they rank as the first prevalent endocrine cancers in human. According to the results of published studies it has been shown the gradual progress from normal to the neoplastic cell in the process of tumor formation is the result of sequential genetic events. Among them we may point the mutations and rearrangements occurred in a group of proto-oncogenes, transcription factors and metastasis elements such as *P53*, *RAS,RET*,*BRAF*, *PPARγ* and Fascin. In the present article,we reviewed the most important essential genes in thyroid cancers, the role of epithelial mesenchymal transition and Fascin has been highlighted in this paper.

## Introduction

Thyroid cancer is considered as a rare malignancy which accounts for 1 to 2% of different type cancers. The annual occurrence rate of thyroid cancer in different parts of the world is reported to be about 0.5 to 10 in every 100,000 persons; yet, this cancer is considered as the most prevalent malignancies of the endocrine system [1–3]. Various studies indicate that women are 2–4 times more likely to suffer from the cancer [4]. There is no difference between the incidence of thyroid cancer between men and women before menarche and after menopause, suggesting that the increased risk of developing thyroid neoplasia in women at child bearing age could be secondary to the effects of estrogen or other pregnancy-related factors. Moreover, the rate of developing thyroid cancers increases significantly after pregnancy, especially, in the last four months of pregnancy. However, it should be noted that, those consuming contraceptive pills or other external sources of estrogen are not at an increased risk of developing thyroid cancers [5]. Positive family history of thyroid cancer is reported in 3-5% of the affected cases [3, 6, 7]. Apart from gender and genetic factors, the body size [8], race [9], geographical distribution [10, 11] and the amount of consumed iodine [12] also influence the risk of developing thyroid cancers. While thyroid cancer is reported at any age, the risk of developing the malignancy increases after the age of 30. Many believe thyroid cancer becomes more invasive with aging [13].

In the majority of the cases, cancer presents as asymptomatic thyroid nodules. In certain cases, however, patients complaint of pain in the neck, lymphadenopathy, and bone and lung involvement.

While thyroid nodules are found in approximately 10% of the people in each society, only 4-5% of the reported cold nodules are malignant [3, 14]. In countries with iodine deficiency,where iodine prophylaxisis common, palpable thyroid nodules are reported in 4-5% of the population [15]. Although more than 90% of thyroid nodules in the general population are benign, some of these nodules might be malignant in nature as about 0.4% of the entire mortalities from cancer occur in those with thyroid malignancies [13].

Pathological analysis of thyroid cancers indicates that four types of thyroid cancers, namely papillary, follicular, anaplastic and medullary thyroid cancers, are more prevalent. The first three are of the follicular cell origin, whereas the medullary carcinoma originates from para-follicular cells (C Cells) [2, 16].

### Papillary thyroid carcinoma (PTC)

Papillary carcinoma is the most prevalent type of thyroid cancer, reported in about 70% of those with thyroid malignancies. Half of the cases present before the age of 40; the remaining is mainly diagnosed in the 6th and 7th decades of life. The cancer is 2–3 times more frequently reported in women. High rates of this type of thyroid cancer are seen in those exposed to high dose X-radiation during childhood. The cases of sporadic thyroid cancer account for 95% of the sufferers and only 5% of the cases are hereditary [17].

Lymph node involvement is common in papillary carcinoma, with metastasis to neck lymph nodes and contiguous tissues reported in 50% and 25% of the cases, respectively. Compared to other thyroid carcinomas, metastasis via blood and particularly lung involvement is more prevalent in papillary carcinoma. This comes while involvement of the bones of the central nervous system and other organs is also possible. With respect to prognosis, papillary carcinomas tend to grow very slowly compared with other types of thyroid cancer. In other words, over 90% of the sufferers now survive for more than ten years; in 80% of them the life span is about 20 years [18].

### Follicular thyroid carcinoma (FTC)

Follicular carcinomas are less prevalent than the papillary type and account for only 15% of thyroid malignancies. Most follicular carcinomas manifest in old ages and after the age of 52. The risk is 2–5 times higher in women. The cancer mainly presents as single cold nodules. In this stage, the lymph nodes are not yet involved. This comes while distant metastasis in lungs and bones are common in many sufferers [19]. Compared with papillary carcinoma, follicular carcinoma has a poorer prognosis, with only 30% of the sufferers living for up to 10 years after the primary treatment [18].

### Anaplastic thyroid carcinoma (ATC)

This carcinoma accounts for about 2% to 5% of thyroid malignancies. It is considered as a highly aggressive and lethal thyroid carcinoma. It can be seen at any age; however, it is more common among those aged between 60 and 70. Women are 5 times more vulnerable of developing the cancer [20]. In 50% of the cases, anaplastic carcinoma may occur following a long-term history of goiter, thyroid adenoma, papillary or follicular carcinoma; its risk of becoming malignant, however, is rather low.

The cancer mainly presents with a rapidly growing neck mass [21]. The malignancy has the poorest prognosis among primary thyroid neoplasms as the afflicted subjects die between 6 to 8 months after diagnosis. The tumor is treatable if diagnosed and treated in early stages [22].

### Medullary thyroid carcinoma (MTC)

Medullary carcinoma accounts for 10% of thyroid malignancies. It is the most invasive type of cancer, on which iodine and chemotherapy are not effective. Surgery remains the only way to treat such cases; however, in cases experiencing recurrence, no successful treatment has been reported. On average, 65% of the patients may survive for 10 years [23].

The cancer is mainly characterized with calcitonin secretion. The cases of sporadic thyroid cancer account for 80% of the sufferers. Medullary Thyroid Carcinoma mainly occurs in the 5th and 6th decades of life and in 75% to 95% of the cases present as a single nodule. Unlike papillary, follicular and anaplastic carcinomas that originate from follicular cells, medullary carcinomas are from para-follicular cells (C-cells), located at the junction of the upper third and the lower two-third of the thyroid lobes [24]. The majority of such tumors therefore are observed in this area. In 50% of the patients, latero-cervical lymph nodes are involved. In 15% of the patients, the symptoms of pressure imposed on esophagus and upper parts of the pulmonary system are reported. Metastasis is reported in 5% of the patients.

Age at the time of diagnosis is the most important diagnostic factor in these patients.

In 20% of the cases, genetic is the most important predisposing factor and medullary cancer is mainly known as an autosomal dominant disorder [25]. Three kinds of hereditary medullary thyroid cancers are known:

Multiple Endocrine Neoplasia 2A (MEN 2A)

Multiple Endocrine Neoplasia 2B (MEN 2B)

Familial Medullary Thyroid Carcinoma (FMTC)

### Most important genetic factors involved in different types of thyroid malignancies

Molecular mechanisms involved in the development of such malignancies are not well known. This comes while existing studies in this regard have reported that the occurrence of 6 or 7 mutations in certain proto-oncogenes during a period of 20 to 40 years is necessary to induce tumor growth [26].

Two families of genes are involved in the proliferation of healthy cells: Proto-oncogenes increase cellular proliferation and tumor suppressor genes stop cell division. The development of thyroid tumors is reported to be secondary to the activation of mutated oncogenes or suppression of tumor suppressor genes or both [27].

### P53

P53 is a tumor suppressor gene, which regulates physiologic cell growth through inducing G1-phase cell cycle arrest [28]. Point mutation of this gene is considered as the most prevalent change linked with such tumors, particularly anaplastic thyroid carcinomas [29, 30]. The presence of these mutations determine tumor invasion. Freeman et al. reported that any changes in the expression of P53 is associated with thyroid cancer; therefore, measuring the rate of P53 expression can be considered as a diagnostic marker in identifying invasive tumors and thus patients with poor prognosis [29]. P53 mutations are reported in 11.1% of patients with papillary carcinoma, 14.3% with follicular carcinoma and 63% with anaplastic carcinoma (in some studies mentioned to be between 75 to 83.3%). Considering the fact that P53 mutation or increased expression of the protein is more common in anaplastic carcinoma compared with other differentiated carcinomas, it could be concluded that the change in the expression rate may influence the transformation of differentiated carcinomas into the anaplasticones. Therefore, studying the expression of p53 can help identify indistinguishable thyroid cancers [31].

### RAS

#### RAS family consists of three genes

- N-RAS on Chromosome 1
- H-RAS on Chromosome 11
- K-RAS on Chromosome 12

These genes are linked to the synthesis of a group of 21 kDa proteins [32] that play an important role in cell growth and differentiation. Point mutation in any of these genes may result in the transformation of proto-oncogenes to oncogenes, and consequently the development of cancer [33]. In more than 30% of human tumors, mutation is reported in the 12th, 13th and 61th codons of RAS proto-oncogene [34]. It is to be noted that mutation in a given allele of these genes is sufficient for the activation of proto-oncogenes [33]. The activation of RAS oncogenes is reported in many of benign and malignant thyroid tumors [35]. Despite the fact that there are a few such tumors, the prevalence of RAS mutations in thyroid neoplasia, particularly follicular and invasive cancers, seems to be high [36]. This comes while no significant correlation has been reported between increased RAS protein expression and the higher rate of distinction or metastasis [37]. RAS proto-oncogene mutations are reported in 20% to 60% of thyroid tumors, especially in follicular cancers [36]. Such mutations are also more prevalent in areas where there is little iodine in the diet [38]. Activated RAS protein is reported in 20% of patients suffering from papillary carcinoma, 53% of those with follicular carcinoma and 6 to 50% of subjects diagnosed with anaplastic carcinoma [39–44].

### RET

Pericentric inversion of chromosome 10 involving the RET (ret proto-oncogene) gene at chromosome 10q11 is known to increase expression of the RET gene. The activation of this oncogene also encodes tyrosine kinase receptor [45].

RET was first discovered by Fusco et al. The activation of the gene is reported in 25% of patients with papillary thyroid cancer [46]. Papillary thyroid cancer is characterized by chromosal rearrangements, through which the promoter and the primary sequences of a genes (R1α- NcoA4- RFG5- hTIF1- CCDC6- RFG7/hTIFR) are transferred to the terminal sequences of the RET gene, developing a fusion gene. RET/PTC fusion gene encodes a permanently active receptor [47]. Many believe these rearrangements are the initiator of tumor formation in individuals with papillary carcinoma [48]. In other words, the expression of RET/PTC oncogene is common in papillary thyroid cancer cells (77% in concealed papillary carcinoma vs. 47% in apparent papillary carcinoma) [49]. RET proto-oncogene mutations are also connected with syndromes with dominant inheritance such as MEN 2A, MEN 2B and familial medullary thyroid cancer.

Nowadays, RET point mutations are applied as markers for identifying hereditary and sporadic Medullary Thyroid Carcinoma (MTC) [50, 51]. In FMTC and MEN 2A, the well-known mutations in exon 10 (cordons 609, 611, 618 and 620) or 11 (codon 634) entangles the extra-cellular area of RET receptor [52, 53].

Duplication/insertion mutations in exon 11 are reported in rare cases of MEN 2A [54, 55]. RET germ line mutations are also reported in exon 13 (cordons 768, 790 and 791), 14 (codons 804 and 844) and 15 (codon 891) of patients with FMTC [56]. While a missense mutation in codon 918 is reported in more than 90% of patients with MEN 2B, mutation at codon 881 in exon 15is a rare finding in these patients [57]. Such variants are mainly linked with the phenotype of MEN 2B [58]. The accurate prevalence of RET/PTC rearrangement is not well known, as the variant is seen in 2.5 to 34.5% of these patients [59].

Somatic changes of RET proto-oncogene are also discovered in 30 to 60% of sporadic PTC tumors, but rarely in familial cases [60]. The abovementioned rearrangements are the only genetic variants reported in PTC patients [49].

### BRAF

In mammalian cells, three isoforms of serine threonine kinase RAF, including ARAF, BRAF and CRAF (RAF1) with different tissue expression rates, are reported [61]. BRAF, located on chromosome7 (7q34), is responsible for controlling cell proliferation and differentiation through the MAP kinase pathway [62]. Inappropriate and abnormal activity of such a pathway may result in a pro-mitogenic force, causing abnormal distinction and proliferation of many human cancers [63]. BRAF mutations are the most prevalent genetic abnormality reported in papillary thyroid carcinoma. It is, however, reported in up to 50% of individuals with anaplastic carcinoma. In about 95% of the cases, mutation of nucleotide 1799, results in the substitution of valin with glutamate at residue 600 (V600E). Such a mutation, however, is not observed in distinguishable follicular and medullary neoplasia sufferers [64–67]. Abundance of BRAF mutations in papillary thyroid carcinoma puts forward this matter that suppressing the activity of BRAF can help develop new treatment modalities for the disease [68].

Nambaet al pointed out the link between BRAF mutation and papillary thyroid carcinoma [69]. Similarly, Webb et al. reported the involvement of MAPK/MEK/RAF pathway in metastasis and tumor growth [70]. Such results suggest that the analysis of BRAF mutations may pave the way for the early diagnosis of patients with papillary carcinoma [69].

Simultaneous mutation in RAS and BRAF has never been reported. This finding is in line with data extracted from other tumor models [71]. Moreover, considering the fact that RAS proto-oncogene mutation is only reported in 20% of patients with papillary carcinoma, studying the simultaneous occurrence of RAS and BRAF mutation is not of much importance [72]. However, further studies are needed to determine the importance of RET/PTC and BRAF mutations in tumorigenesis [71].

### PPARγ

For the first time in 2000, Kroll et al. showed the rearrangement of PAX8 and PPARγ in individuals with follicular thyroid carcinoma [73]. PPARγ (Proxisome Proliferator Activated Receptor Gamma) is a transcription factor involved in lipid metabolism and cell differentiation [74]. PAX8 (Paired Box Gene 8), the transcription factor in thyroid tissues, controls expression of certain proteins such as TSHR, thyroid peroxidase, and Sodium-Iodine transporter [75]. Protein arising from such fusion (PAX8/PPARγ) has the same function as that of PPARγ; however, its characteristics are different from PAX8 and PPARγ [76]. Recent studies in this field demonstrated PAX8/PPARγ rearrangement in 25%-63% of patients with follicular carcinoma [77] and 37.5% of those suffering from papillary carcinoma [78]. (Table 1).

**Table 1:** **Most prevalent mutations noted in different types of thyroid cancers**

Thyroid tumor type	Mutation				Rearrangement		References
	***P53***	***RAS***	***RET***	***BRAF***	***RET/PTC***	***Pax8/PPARγ***	
Papillary Carcinoma	+	+	-	+	+	+	[31, 39, 43, 44, 48, 49, 51, 64–68],[78]
Follicular Carcinoma	+	+	-	-	-	+	[31, 36, 41, 44, 48, 64–67, 77]
Anaplastic Carcinoma	+	+	-	+	-	-	[31, 36, 43, 48, 64–66, 73]
Medullary Carcinoma	-	-	+	-	-	-	[31, 36, 47, 48, 50, 52–58, 73]

### Metastasis

Metastasis is a complex biological process that requires cancer cells to be separated from adjacent cells in a way that they could target the extracellular matrix (ECM) and the basilar membrane, and enter blood circulation. Such cells escape the immune system and reach farther tissues, resulting in the formation of secondary tumors at other locations. The process is associated with increased risk of death from cancer. Much research has been conducted to study the pathology and treatment of metastasis, especially, with respect to molecular mechanisms. Epithelial mesenchymal transition (EMT) is an important part of the metastatic cascade [79]. They initiate metastasis and thus have attracted many researchers studying cancer and metastasis.

Fascin, as one of the proteins involved in metastasis, is very important in such a process. In mammalians, Fascin has three isoforms:

◦ Fascin-1, specific for the mesenchyme and neural system, is situated at 7p22 [80].

◦ Fascin-2 islimited to photo-receptor cells in the eye. Its encoding gene is in 17q25 [81].

◦ Fascin-3 found in testis. Its locus is in 7q31 [82].

From among the mentioned proteins, Fascin-1 binds to actin filaments and may form cellular protrusions such as filopodia and lamelipodia. The formation of such structures can result in higher rate of invasion and metastasis [83]. Fascin-1 is a genetically conserved protein (493 amino acids, 55KD), linked with actin filaments in the cytoplasm from two different sites. Through arranging these filaments, Fascin-1 can organize cell movement [84]. Immunohistochemistry (IHC) and Tissue Microarray (TMA) studies of various cancers, especially metastatic lung and pancreatic cancer, have revealed increased expression of Fascin-1 in cancerous tissues cells. For instance, healthy lung cells have no Fascin-1. This comes while IHC studies showed increased expression of Fascin-1 in 89% of such cells if they become cancerous and in the early stages of the disease. This is also true for pancreatic cancer. IHC studies showed that from among 57 persons suffering from pancreatic cancer, 95% had increased Fascin-1 expression. In another study, conducted two years later, TMA studies on 68 people suffering from this cancer showed increased expression of such protein in 97% of the cases.

IHC studies have confirmed increased Fascin-1 expression in papillary, follicular and anaplastic thyroid cancer. On the other hand, the expression of such protein is not reported in healthy individuals and those suffering from goiter [85]. Despite the outstanding role of Fascin-1 in increasing the invasion and cellular movement, available studies have failed to link any genetic mutation with the increased expression responsible for the characteristic. The regulatory region of these genes consists of 250 base pairs and is located at the 5’-flanging end of the gene [86]. Therefore, studying the genetic changes of the promoter region of fascin-1 as one of the factors involved in the regulation of gene expression and discovering the relation between such mutations and the rate of metastasis can pave the way for early diagnosis of those suffering from metastatic thyroid cancer.

## Conclusion

In the past decade, many studies have been conducted on genetic changes and molecular biology of thyroid cancer to improves the accuracy of diagnosis and the effectiveness of treatment modalities. From among them the mutations and rearrangements of certain proto-oncogenes as well transcription and metastatic factors such as P53, RAS, RET, BRAF, PPARγ and Fascin are of great importance. In view of the fact that the available methods are not capable of diagnosing thyroid cancer in early stages, detection of differentiated mutations may be an effective method in this regard. It seems that in various cancerous cells, there are different and very special mechanisms for metastasis processes. Genetic studies can also help identify the reason behind changes noted in the expression of suppressor genes or metastatic activators in order to find effective solutions to prevent malignant variants of these cancers.

## Abbreviations

PTC: Papillary thyroid carcinoma
FTC: Follicular thyroid carcinoma
ATC: Anaplastic thyroid carcinoma
MTC: Medullay thyroid carcinoma
MEN 2A: Multiple endocrine neoplasia 2A
MEN 2B: Multiple endocrine neoplasia 2B
FMTC: Familial medullary thyroid carcinoma
PPARγ: Proxisome proliferator activated receptor gamma
PAX8: Paired box gene 8
ECM: Extra cellular matrix
EMT: Epithelial mesenchymal transition
IHC: Immunohistochemistry
TMA: Tissue microarray
MAPK: Mitogen- activated protein kinase.
